# Active Service and Medical Officers' Pensions

**Published:** 1915-03-13

**Authors:** 


### ACTIVE SERVICE AND MEDICAL OFFICERS' PENSIONS.

The position in respect to superannuation of the medical and other officers of the various public services who are at present engaged on naval and military service has given rise to much discussion among those chiefly concerned. As regards the Mental Hospital Service, the Home Office, after consultation with the Local Government Board, has intimated that it will not object to any Asylum Visiting Committee, if it so desires, treating active service during the present war as a part of the officer's asylum service, and as pensionable under the Asylum Officers' Superannuation Act, 1909. It may be inferred that the Local Government Board will be guided by the same principle in dealing with similar requests under the Poor-Law Officers' Superannuation Act, 1896.

The pronouncement of the Home Office does not leave matters in an entirely satisfactory condition. The powers of public bodies under the various Superannuation Acts are limited by statute, and any expenditure under them, not legally authorised, may be objected to in the Courts. In the case of the elementary school teachers and of the police this difficulty has been met by the passage last year of special Acts providing that service in the war is to be equivalent for superannuation purposes to service as a teacher or in the police force. Similar Acts are required in the case of other public services, not only to make the legal position perfectly clear, but also to ensure uniformity of treatment. As matters stand, no officer who volunteers for active service will be entitled, as a matter of right, to have the period of his enlistment reckoned in his years of service for superannuation purposes. The decision to do so will be an act of grace on the part of the public body of which he is an officer. Although the majority may be trusted to act generously, protection is required against the possibility of injustice.

Another matter which requires consideration is the provision to be made in the case of an officer who is killed or becomes permanently incapacitated, while on active service, from performing the duties of his civilian office. In a few cases the Local Government Board has already sanctioned special payments being made to the dependants of officers of various local bodies who have been killed in the war, such payments consisting in the return of the superannuation deductions, together with a gratuity, or in the payment of the full salary for a period of some weeks after the death. Special consideration of this kind, however, should be universal, and fixed by definite regulations. As regards officers permanently incapacitated, the National Poor-Law Officers' Association suggests that the superannuation allowance should be calculated on the salary and emoluments the officer would have been receiving had he not joined the naval or military forces.

This suggestion seems eminently reasonable. The superannuation allowance would, of course, be additional to any naval or military pension the officer might receive. The title to superannuation under any of the Acts depends upon the fact of incapacity, and is unaffected by the way in which that incapacity is brought about, or by the fact that the officer may be entitled to compensation for injuries he may have received. It is necessary to insist upon this because it is possible that in the future some public bodies may try to reduce the superannuation allowance to which one of its officers is legally entitled, on the ground that he is the recipient of a Navy or Army pension for the incapacity necessitating his retirement.

